# Introducing *Open Biology*

**DOI:** 10.1098/rsob.110001

**Published:** 2011-09

**Authors:** David Glover, Christine Holt, Louise Johnson, Peter Parham

## Why is the Royal Society launching *Open Biology*?

Since its creation the Royal Society has endeavoured to represent and support science in the broadest sense. Providing venues for scientists to publish their research is an integral part of this activity.

The Society's established research journals *Proceedings B* and *Biology Letters* are very strong in ecology, evolution and behaviour and in overlapping areas. However, they publish very little “fundamental” research in the areas of cell biology, developmental and structural biology, molecular biology, biochemistry, neuroscience, immunology, microbiology and genetics.

The Society has therefore decided to launch this new journal, *Open Biology*, to provide a Royal Society publishing venue for researchers working in biology at the molecular and cellular level.

## What is *Open Biology*?

*Open Biology* is the Society's first wholly open access and online-only journal.

It will publish original, high quality, research in the areas outlined above. It will be overseen by a team of Subject Editors and an international Editorial Board all of whom are active scientists. It will draw on the high standards maintained by the Society over its 350 year tradition in scientific publishing.

The journal complements the Royal Society's existing optional open access journals and reinforces open access-friendly policies. The funding required to make *Open Biology* open access will derive from article processing charges, which will cover the expenses associated with peer review, composition, hosting, and archiving.

## How will the journal operate?

The editorial team will be responsible for developing the editorial direction of the journal and will be the final authority on what is published. The journal will be run with the full support of the Society's Editorial Office and the Editorial Board. *Open Biology* will be published online on a continuous publication model where articles are immediately citable. Online archiving and information on article downloads will be available. Articles will be published under the terms of the Creative Commons Attribution Licence, leaving copyright with the authors, but allowing anyone to download, reuse, reprint, modify, distribute, and/or copy articles provided the original authors and source are cited.

Our vision is to provide a high quality publishing venue for biology at the molecular and cellular level. We urge you to submit your very best research to *Open Biology*. In return we will provide the peer-review, high quality editorial feedback, rapid publication and international dissemination of your research associated with all Royal Society journals.

